# Financial performance of people with affective disorders: A prospective, European-wide study

**DOI:** 10.1192/j.eurpsy.2025.468

**Published:** 2025-08-26

**Authors:** T. Jiang, G. Gaastra, J. Koerts

**Affiliations:** 1 Department of Clinical and Developmental Neuropsychology, University of Groningen, Groningen, Netherlands

## Abstract

**Introduction:**

Financial capability, which encompasses both financial competence and financial performance, is a key requirement for an autonomous and independent life. However, individuals with psychiatric disorders often report difficulties with financial capability and financial stress.

**Objectives:**

The current study aims to disclose the impact of affective disorders (AD) on financial performance using prospective data from wave 8 and wave 9 of the Survey of Health, Retirement and Ageing in Europe (SHARE).

**Methods:**

The SHARE project includes individuals born on or before 1971 or households with at least one member meeting this age criterion. During each wave, participants reported whether they received one of 17 diagnoses, including “affective or emotional disorders, including anxiety, nervous or psychiatric problems”. Furthermore, monthly household net income and whether participants experienced difficulties in managing money, challenges in making ends meet, and their debt situation were recorded. The differences between the AD and a control group (i.e., participants without affective or neurological conditions) on monthly household net income and the three financial performances were examined for both waves. Logistic regression analyses were performed to analyze whether an AD diagnosis predicted financial performances in wave 8 and wave 9.

**Results:**

2,645 individuals reported an AD diagnosis in wave 8, and 47,068 were classified as controls. In wave 9, 3,574 individuals indicated having an AD diagnosis and 60,902 were classified as controls. In both waves, individuals with AD received a lower monthly household net income and reported more difficulties across all three financial performances relative to controls. Furthermore, in both waves, AD individuals reported having more debts on cars and other vehicles, debt on credit/store cards, loans, debts to relatives or friends, overdue bills, and other types of debts than controls. In wave 8, the odds of experiencing difficulties in managing money, making ends meet, and having debts were 3.61, 1.45, and 1.73 times higher, respectively, in AD individuals than in controls. A diagnosis of AD increased the likelihood of future (i.e., during wave 9) financial difficulties: 2.67, 1.38 and 1.68 times for managing money, making ends meet, and having debt, respectively.

**Conclusions:**

Individuals with AD are more prone to experiencing impairments with both current and future financial performance and might face financial difficulties. The present study emphasizes the importance of recognizing financial difficulties in individuals with AD and offering financial assistance when necessary, especially since financial difficulties might exacerbate affective symptoms.

**Disclosure of Interest:**

None Declared

